# The Effect of Relative Humidity on Creep Behavior of Cement Paste Microprism †

**DOI:** 10.3390/ma18020406

**Published:** 2025-01-16

**Authors:** Zhao Chen, Mahdiar Dargahi, Luca Sorelli

**Affiliations:** 1 Institute of Mechanics, Chinese Academy of Sciences, Beijing 100190, China; 2 Department of Civil Engineering, Laval University, Québec City, QC G1V 0A6, Canada; mahdiar.dargahi.1@ulaval.ca

**Keywords:** cement paste, microprism, relative humidity, creep modeling, foundation effect, analytical modeling, finite element method

## Abstract

Despite decades of extensive studies, the mechanism of concrete creep remains a subject of debate, mainly due to the complex nature of cement microstructure. This complexity is further amplified by the interplay between water and the cement microstructure. The present study aimed to better understand the creep mechanism through creep tests on microprisms of cement paste at hygral equilibrium. First, microprisms with dimensions of 150 mm × 150 mm × 300 mm were prepared by precision cutting from a cement paste specimen with a water-to-cement ratio of 0.4. Subsequently, uniaxial compression and creep tests were carried out on these microprisms in a chamber with controlled relative humidity (RH). To mitigate the impact of plasticity and damage, the applied peak load was set to generate a stress level that was approximately 40% of the compressive strength. Moreover, an analytical coefficient φ was formulated to account for the foundation effect on microprism creep, agreeing with the numerical analysis employing the finite element method. Our findings showed that the microscale creep compliance varied when the RH level was changed from 90% to 11%. Furthermore, logarithmic and power-law models were both applied to simulate creep curves. Lastly, the modeled creep behaviors were compared with those obtained by microindentation experiments in previous studies.

## 1. Introduction

Concrete creep is a critical factor for the long-term safety and performance of concrete structures, particularly in large-span bridges [1]. A study conducted by Bažant et al. [2] highlighted that 56 prestressed concrete bridges with large spans have experienced considerable long-term deflections, beyond the predictions of current design standards, including the ACI Committee 209, CEB-fib, and GL models. Notably, the long-term concrete creep exhibited a logarithmic evolution after three years, with no indication of a definitive upper limit.

After a century of research, the intricate nature of concrete creep remains a great challenge, largely due to its multiscale characteristics ranging from the nanoscale to the macroscale [3]. Moreover, it is further complicated by the heterogeneous microstructure of concrete, which includes components like calcium silicate hydrates (C-S-H), calcium hydroxide (CH), unhydrated clinker, voids, interfacial transition zones (ITZs), and more [4,5,6,7]. It has been well-established that concrete creep is primarily determined by the C-S-H microstructure [8,9,10], which typically accounts for 50–60% of hardened cement paste in volume [7]. Feldman and Sereda [11] proposed that the concrete creep phenomenon is caused by the sliding of the C-S-H layered microstructure and the reconfiguration of inter-particle bonds. Their assumption was later validated by the three-point bending relaxation tests performed on synthetic C-S-H [12]. According to Jennings’ colloid model, cementitious materials contain two distinct types of C-S-H: low-density (LD) C-S-H and high-density (HD) C-S-H [13,14], which were evidenced by a statistical nanoindentation study [5]. Furthermore, Vandamme and Ulm [15] linked the concrete creep to the nanogranular behavior of these C-S-H phases, which show different packing densities: 0.69 for LD C-S-H and 0.78 for HD C-S-H.

Additionally, the complex interplay between the C-S-H microstructure and internal water increases the difficulty of understanding and predicting concrete creep behavior [16,17,18,19]. According to Jennings’ colloid model [14], the interlayer water, the gel pore water, and the capillary water within the C-S-H are progressively filled as the relative humidity (RH) level increases from 0% to 100%. At the macroscale, Wittmann [20] performed a creep experiment on a cement paste cylinder with a diameter of 18 mm and a height of 60 mm, showing the enhancing effect of increased RH levels ranging from 0% to 98%. However, achieving internal water equilibrium takes at least three months at various RH levels. At the nanoscale, a similar enhancing effect of RH was recently observed in the nanoindentation relaxation behavior of C-S-H as the RH level was increased from 33% to 86% [21]. Notably, the hygral equilibrium was reached within one day, significantly reducing the testing time. Furthermore, a transition from short-term to long-term creep was observed within the first few seconds: the short-term creep mechanism is likely due to the water microdiffusion phenomenon, initially proposed by Powers [22,23] and later experimentally confirmed using a ^1^H NMR technique [24]; whereas, long-term creep is facilitated by the interlayer water within the sliding C-S-H sheets.

To address the aforementioned concerns, several advanced testing methods have been developed in recent years to explore microscale creep in cementitious materials:

1. Microindentation: This method has been effectively used to examine the effects of relative humidity (RH) on the logarithmic creep behavior of concrete materials [18,25,26,27]. However, it is noteworthy that the pressure generated under the Berkovich tip can be substantial, reaching hundreds of megapascal for a penetration depth of 20~40 mm. To precisely extract the creep property, one must take into account the additional plastic effect [28] or employ a spherical tip to mitigate stress concentration when employing linear viscoelastic modeling [29].
2. Microbeam: Recent work proposed to use micro-dicing saws to generate micro-cantilever beams from hardened cement paste, measuring 300 × 300 × 1650 µm^3^ [30,31]. The bending creep and creep recovery were evaluated using a power-law of creep compliance. The authors argue that the microscale creep behavior of cement paste is qualitatively and quantitatively consistent with its macroscopic creep. Nevertheless, it is important to note that the RH level was not controlled during these experiments.
3. Micropillar (or microprism): The majority of studies on creep behavior have employed cement micropillars for uniaxial compression experiments aimed at examining compressive strength and Young’s modulus [32,33,34,35]. Notably, a recent study [36] employed focused ion beam (FIB) milling to generate cement micropillars, with diameters of 0.5 μm and 5 μm, which were used to capture the creep behavior of C-S-H. It is argued that the creep compliance of C-S-H follows a power-law relationship rather than a logarithmic one. Note that their experiment was conducted at a controlled temperature of 24.4 °C ± 0.5 °C and a constant RH level of 39% ± 3%.

Microprisms (or micropillars) provide a significant advantage over other experimental techniques by enabling uniaxial compression creep tests. The linear concrete creep behavior can be controlled at stress levels ranging from 30% to 50% of the compressive strength, which can be modeled using uniaxial creep compliance [36,37]. However, the foundation effect—a portion of the recorded deformation attributed to the substrate—must be accounted for to accurately isolate the creep behavior of a microprism. For axisymmetric punches, such as circular pillars, Sneddon’s analytical solution can be adopted to simplify the modeling of the foundation effect by relating the penetration load to the depth [33,34,36,38]. In contrast, for non-axisymmetric punches, such as microprisms with square cross-sections, numerical methods are required, as the foundation effect cannot be analytically simplified. While the foundation effect can be reduced experimentally, such as by cutting a few micrometers of the cement specimen into a glass substrate bonded to its bottom [32], further theoretical investigation is still needed to effectively remove this effect.

To the best of the authors’ knowledge, research on microscale uniaxial creep of cementitious materials at different RH levels has not been reported previously. Furthermore, developing an accurate and robust creep model is essential for predicting the long-term performance of concrete materials and structures. However, this topic continues to be a subject of ongoing discussion at both the microscale and macroscale levels. For instance, creep models in design codes like CEB and ACI employ power-law functions, whereas the *fib* Model Code 2010 adopts a logarithmic function [39]. Therefore, the present study employs a state-of-the-art micromechanical approach to address the following questions: (i) How does the internal water affect the uniaxial compression creep of cement microstructures? (ii) Is the microscale creep behavior of cement paste determined by logarithmic or power-law creep compliance? This study aimed to better understand the creep behavior of cement microstructure, providing a basis for designing concrete materials and structures with enhanced resilience to long-term deformations.

## 2. Materials and Methods

### 2.1. Material Preparation

In this study, we used a general-purpose cement (GU) provided by CRH Cement (Québec, QC, Canada). The paste was formulated by blending the cement with water in a high-shear mixer (water-to-cement ratio (w/c) = 0.4). The mixture was then cast into a cylindrical tube with a diameter of 30 mm. After 24 h of hydration, the specimen was demolded, placed in a sealed tube with a diameter of 34 mm, filled with water at 20 °C, and cured for 6 months before testing.

### 2.2. Microprism Fabrication

After the curing, a square slice measuring 10 × 10 × 4 mm^3^ was cut from the center of the cement paste cylinder. The slice then underwent a multi-step polishing procedure. Initially, it was coarsely polished using abrasive paper of 240, 400, 600, and 1200 grit. Subsequently, fine polishing was conducted using 6 µm and 1 µm oil-based diamond suspension. After each polishing step, the slice was cleaned with isopropanol in an ultrasonic bath for two minutes. It was then stored in a desiccator for one day to prevent carbonation.

**Figure 1:**
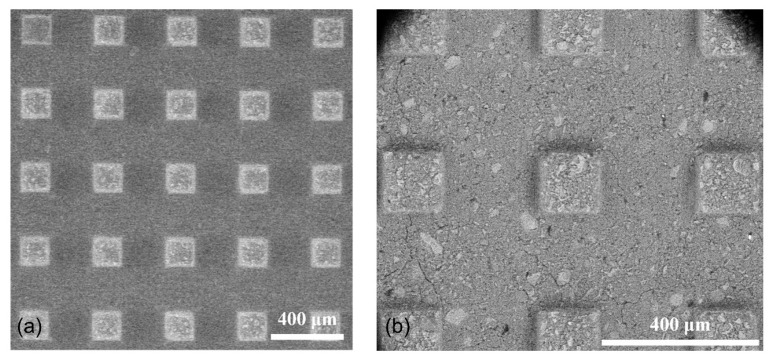
Representative ESEM photos of the matrix microprisms from the top view at two different magnifications: (**a**) 50× and (**b**) 150×.

Next, a high-precision dicing saw (DAD 3350, Disco, Tokyo, Japan) was employed to fabricate a grid of microprisms (150 × 150 × 300 µm^3^) by penetrating 150 µm into the surface in two perpendicular directions. The inter-distance between the two parallel cutting lines, indicated by the diamond blade thickness, was maintained at 250 µm. Following the dicing process, the sample was washed with isopropanol in an ultrasonic bath for 5 min and was then stored for two days in the desiccator before subsequent testing. The quality of the microprisms was examined using an environmental scanning electron microscope (ESEM) at a low vacuum (Figure 1), followed by micromechanical tests.

### 2.3. Experimental Setup

**Figure 2:**
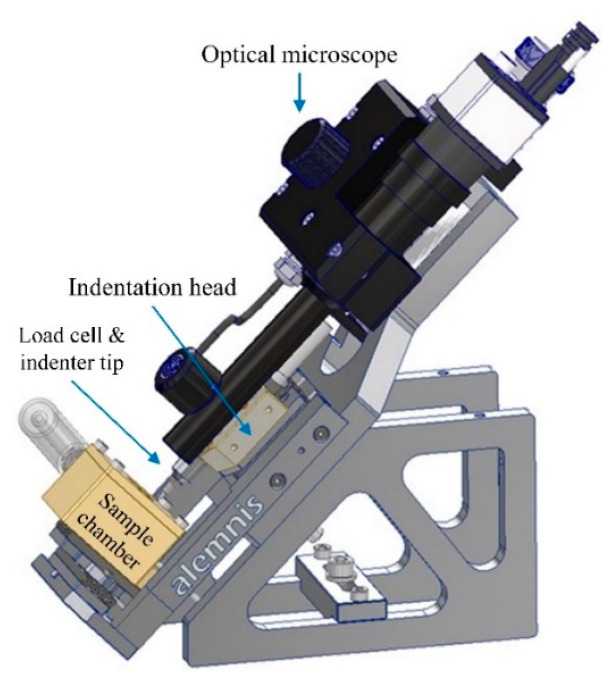
The nano-indenter instrument used in this study.

Micromechanical experiments were performed using a nano-indenter (Alemnis AG in Thun, Switzerland) paired with an environmental sample chamber to precisely control the RH level (from 5% to 95% ± 1.5%), as depicted in Figure 2. The testing temperature was maintained at 23 °C, with an accuracy of 0.1 °C. Before testing, the sample was kept in the humidity chamber for a minimum of 24 h to ensure that the internal moisture level reached equilibrium. Compression and creep experiments were conducted through a drying process by decreasing the RH level from 90% to 11%. A flat punch tip made of diamond, with a diameter of 382 µm, was used to compress the cement microprisms. Before the experiment, thermal drift was measured and calibrated on a reference sample. The compressive tests were performed on 9 microprisms at an RH level of 11% and on 11 microprisms at an RH level of 90%, with a displacement control of 50 nm/s. For the creep experiments, load control was used, with a 5 s loading time to reach the maximum load, followed by a 180 s holding period, and then unloading over 5 s. Due to the difference of compressive strength ($f_{c}$) at various RH levels, the maximum load of 0.6 N and 0.4 N for creep experiments were used for the two RH levels of 11% and 90%, respectively, both generating a stress-to-compressive strength ratio of 0.4, i.e., $\sigma_{0}/f_{c}$ = 0.4. The creep experiments were conducted on 9 microprisms at an RH level of 11% and 13 microprisms at an RH level of 90%.

## 3. Foundation Effect

### 3.1. Analytical Modeling

**Figure 3:**
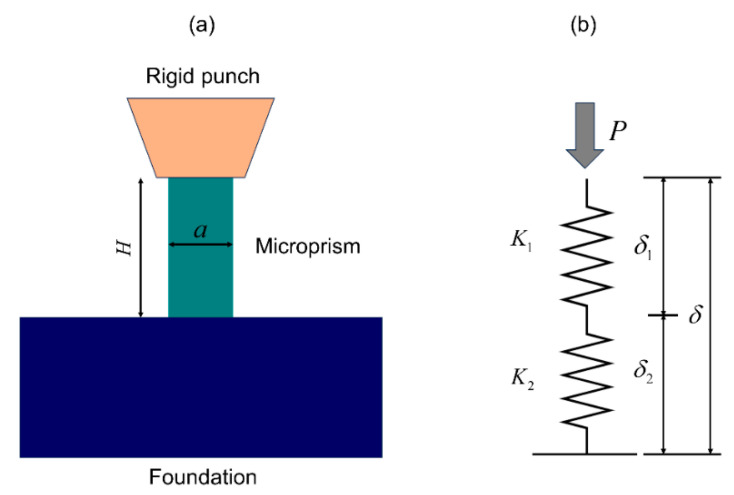
Schematic diagram of interaction between microprism and foundation (**a**) and a simplified mechanical model (**b**).

As illustrated in Figure 3, when a load P was applied to the microprism, the total displacement *d* was recorded by the instrument, which includes two components: displacement $\delta_{1}$ of the microprism and displacement $\delta_{2}$ of the foundation. To correct for the foundation effect from the total displacement, we adopted a simplified model comprising a spring for a microprism with stiffness $k_{1}$, connected in series with another spring for a foundation with stiffness $k_{2}$.

For a square cross-section shaped microprism with a side length of a and a height of H, the stiffness $k_{1}$ is calculated using the following equation:

(1) $$k_{1}=\frac{Ea^{2}}{H}$$

where E indicates the Young’s modulus of both the microprism and foundation. Regarding the foundation effect, it can be understood as a flat-ended punch where the microprism penetrates the foundation. The analytical solution for the foundation stiffness can be found in the work of Sneddon for an axisymmetric circular punch [38], which was used to account for the foundation effect of a circular cement micropillar [34]. Interestingly, for a non-axisymmetric square punch, numerical analysis showed that it agrees well with the circular punch by considering an equivalent area $\pi r_{eq}^{2}=a^{2}$ [40]. The foundation stiffness for this study can thus be written as:

(2) $$k_{2}=\frac{2Er_{eq}}{1-\nu^{2}}=\frac{2Ea}{\sqrt{\pi }(1-\nu^{2})}$$

Furthermore, the relationship between the microprism displacement $\delta_{1}$ (or strain $\varepsilon_{1}$) and the total displacement δ (or strain ε) is given by:

(3) $$\delta_{1}=\varphi \delta ,\;\varepsilon_{1}=\varphi \varepsilon$$

where $\varphi =(1+k_{1}/k_{2}{)}^{-1}$ is a coefficient that accounts for the foundation effect, which is dependent on the ratio a/H. Combining Equations (1) and (2), it can be rewritten as:

(4) $$\varphi =(1+\frac{\sqrt{\pi }a(1-\nu^{2})}{2H}{)}^{-1}$$

The coefficient φ decreases as the ratio a/H  increases. For the microprism in this study, when a/H = 0.5, the coefficient φ is approximately 0.7.

### 3.2. Numerical Modeling

To compare and validate the results of the analytical analysis, a finite element method (FEM) model was developed using ABAQUS 2019. The mechanical properties of the cement microprism and foundation were defined as follows: Young’s modulus of 3.89 GPa (corresponding to the average experimental result at 11% RH) and a Poisson’s ratio of 0.2. The microprism and foundation were meshed using 3D 8-node hexahedral elements (C3D8R). A pressure of 26.67 MPa, equivalent to a concentrated load of 0.6 N, was then applied to the top surface of the microprism. The bottom surface of the foundation was fully constrained to simulate a fixed support. To facilitate the comparison with the analytical method, various FEM models were analyzed for different aspect ratios (a/H): 0.1, 0.3, 0.5, 0.75, 1.0, 1.2, 1.5, 1.76, and 2.0.

**Figure 4:**
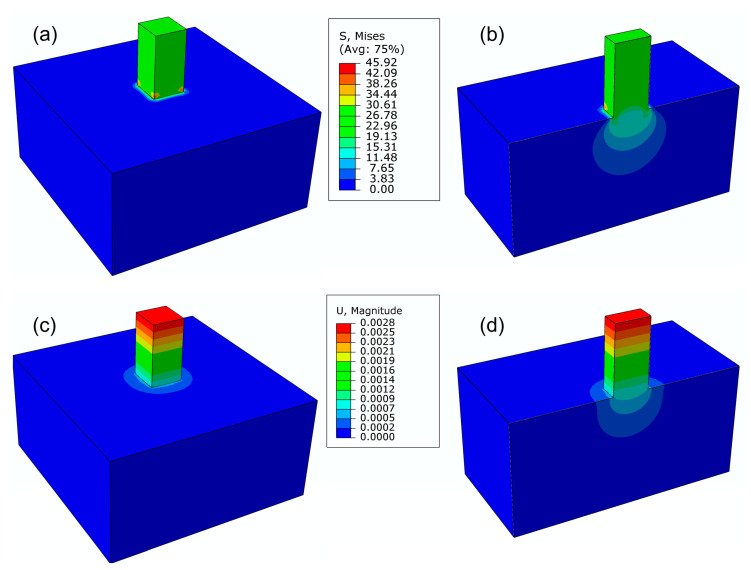
Contour plots of von Mises stress (in MPa) and displacement (in mm): (**a**) full view of von Mises stress, (**b**) sectional view of von Mises stress, (**c**) full view of displacement, and (**d**) sectional view of displacement.

**Figure 5:**
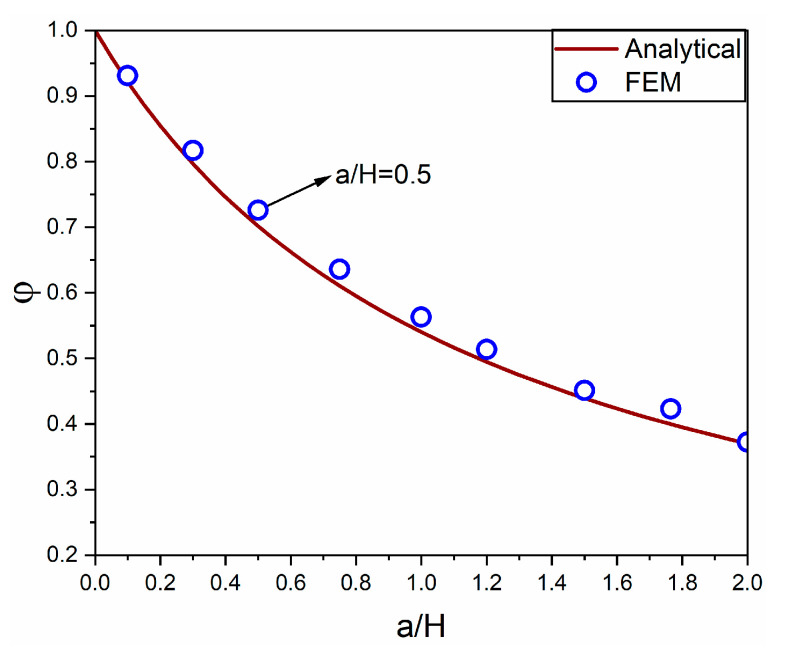
Comparison of the foundation effect coefficient (φ) obtained from the analytical method and the FEM model.

According to the physical interpretation of von Mises theory, yielding starts when the elastic energy of distortion reaches a critical value [41]. The mathematical expression of von Mises stress ($\sigma_{v}$) is written as:

(5) $$\sigma_{v}=\sqrt{\frac{1}{2}\left[(\sigma_{1}{-\sigma_{2})}^{2}+(\sigma_{2}{-\sigma_{3})}^{2}+(\sigma_{3}{-\sigma_{1})}^{2}\right]}$$

where $\sigma_{1}$, $\sigma_{2}$, and $\sigma_{3}$ are the principal stresses. The contour plots of von Mises stress for the cement microprism with a dimension of 150 × 150 × 300 µm^3^ (a/H = 0.5) are presented in Figure 4. The maximum stress (45.92 MPa) occurred around the four bottom corners of the microprism due to the stress concentration. From the sectional view in Figure 4b, a zone as deep as approximately 250 µm beneath the foundation was influenced by the indentation effect of the microprism. Additionally, Figure 4c,d illustrates the displacement of the microprism–foundation system, with the maximum displacement (indicated in red) observed at the top surface of the microprism. The coefficient φ of the FEM model was calculated as 1 minus the ratio of the average displacement of nodes on the top surface of the microprism to the average displacement of nodes on the bottom surface. As shown in Figure 5, the analytical φ slightly underestimated the deformation of the microprism compared to the FEM results. For instance, the analytical coefficient φ is 0.70, while the FEM coefficient φ is 0.72. This discrepancy results from converting the square area into an equivalent circular area by approximating the prism punch indentation as a circular punch indentation. Nevertheless, the discrepancy, with a relative error of 3%, is minor and considered acceptable.

## 4. Creep Modeling

For the uniaxial compression, the shifted creep strain $\varepsilon_{1}\left(t\right)-\varepsilon_{1}\left(0\right)\;$ during the holding phase can be written as:

(6) $$\varepsilon_{1}\left(t\right)-\varepsilon_{1}\left(0\right)=\sigma_{0}(J\left(t\right)-J\left(0\right))$$

where $\sigma_{0}$ is the holding stress applied to the microprism and $J\left(t\right)-J\left(0\right)$ represents the shifted creep compliance, where $J\left(0\right)=1/E_{0}$ is the elastic compliance. To model creep, we first consider a logarithmic creep compliance [18,27,42]:

(7) $$J(t)-J\left(0\right)=\frac{1}{C}ln(1+\frac{t}{\tau_{0}})$$

where C and $\tau_{0}$ are two fitting parameters representing creep modulus and characteristic time, respectively. Specifically, C controls the long-term creep rate, while $\tau_{0}$ indicates the characteristic time required for the creep curve to transition to a logarithmic response. It is noteworthy that this logarithmic creep compliance aligns with the term for the long-term creep of the micro-prestress solidification model [43]. Additionally, we employed a power-law model, previously used to model micropillar creep [36] and micro-cantilever beam creep [30] of cementitious materials, which is written as:

(8) $$J\left(t\right)-J\left(0\right)=\alpha {\left(\frac{t}{t_{1}}\right)}^{\beta }$$

where α and β are fitting parameters. Specifically, α represents the creep compliance at 1 s, and β is the exponent of the power function. $t_{1}$ denotes the unit time, which is equivalent to 1 s.

## 5. Experimental Results

### 5.1. Compression

**Figure 6:**
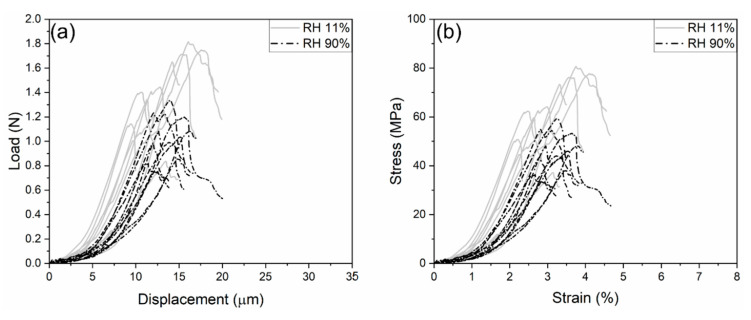
The curves of (**a**) load vs. displacement and (**b**) stress vs. strain for the compression tests at two RH levels.

Figure 6a,b show respectively the load-displacement and stress-strain curves of cement microprisms at RH levels of 11% and 90%. It is noteworthy that the displacement and strain are corrected by the coefficient φ, which accounts for the foundation effect. At the initial loading, the curves show an exponential increase in load, followed by a linear relationship with strain. The load reaches its peak ($P_{max}$, Table 1) within the strain range of 2% to 4%. Then, it is followed by an unloading phase attributed to precise displacement control.

The mean compressive strength, i.e., $f_{c}=P_{max}/a^{2}$, increases from 46.26 MPa to 64.67 MPa, as the RH level decreases from 90% to 11%. Similarly, Young’s modulus (E) increases from 3.01 GPa to 3.89 GPa, which is determined by the slope in the range of 50~80% of $P_{max}$. However, the standard deviations of $f_{c}$ and E are substantial, owing to the heterogeneous nature of cement microstructure. Therefore, an analysis of variance (ANOVA) was performed at a significance level of $\alpha_{0}$ = 0.05 to examine the effect of RH on $f_{c}$ and E. Our results showed that the *p*-values are 0.0015 and 0.013, respectively, which are both lower than $\alpha_{0}$, indicating that the RH effect is significant. Details of the ANOVA results can be found in Appendix A. It is noteworthy that the data for $f_{c}$ and E follow normal distributions, as confirmed by both the Anderson–Darling test and the Shapiro–Wilk test at the significance level of 0.05 [44]. Further details of these statistical analyses are provided in Appendix B.

### 5.2. Creep

**Figure 7:**
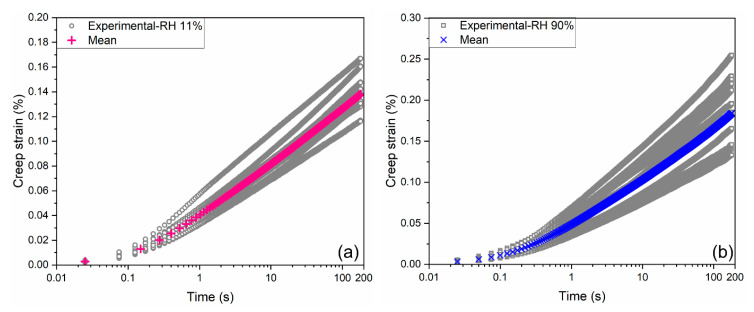
Creep strain of microprisms at RH levels of 11% (**a**) and 90% (**b**).

After correcting the foundation effect with the factor φ, the shifted curves of experimental creep strain ($\varepsilon_{1}\left(t\right)-\varepsilon_{1}\left(0\right)$) in percentage versus semilog time scale are depicted in Figure 7a,b for RH levels of 11% and 90%, respectively, along with the mean curves After a 180 s hold, the average creep strain reaches approximately 0.14% at the RH level of 11%, and 0.18% at the RH level of 90%. Notably, the creep strain increases linearly within the initial few seconds, while elevating in an asymptotically logarithmic fashion afterward.

## 6. Creep Modeling Results

### 6.1. Modeling Results

**Figure 8:**
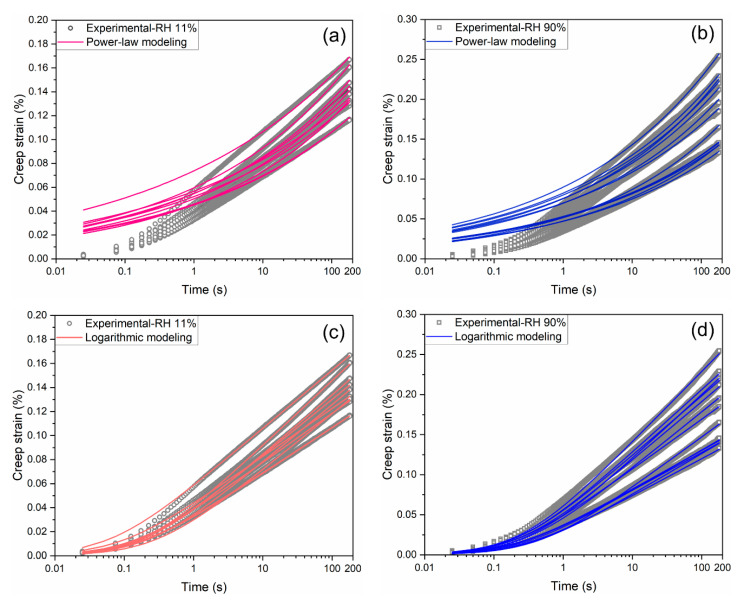
Experimental and modeling results for microprism creep using power-law (**a**,**b**) and logarithmic models (**c**,**d**).

The creep strain curves depicted in Figure 7 are fitted with both logarithmic and power-law creep compliance (Equations (7) and (8)) through a least-square method. The comparisons between the experimental and modeling results are presented in Figure 8. Generally, the logarithmic model demonstrates better capability in capturing the creep behavior at both RH levels, particularly during the first few seconds of creep, as shown in the plots. Moreover, as outlined in Table 2, the coefficient of determination (*R*^2^) for the logarithmic model approaches 1, indicating a highly precise prediction. Table 2 also provides the mean and standard deviation values of the fitting parameters C and $\tau_{0}$ for the logarithmic model, as well as α and β for the power-law model.

In the logarithmic model, the mean creep modulus C, which controls the long-term asymptotic creep rate, increases from 65.6 GPa to 137.8 GPa as the RH level decreases from 90% to 11%. This rise in C suggests an enhancing effect of the internal moisture on the microprism creep behavior. Meanwhile, the mean characteristic time $\tau_{0}$, which determines the initial creep rate, decreases from 0.26 s to 0.17 s with the decreasing RH. In contrast, in the power-law model, the mean value of specific creep compliance α is 0.04 GPa^−1^ at the RH level of 90%, which is double the α value of 0.02 GPa^−1^ at the RH level of 11%. However, the exponent β exhibits similar values of 0.20 and 0.19 at the two different RH levels.

**Figure 9:**
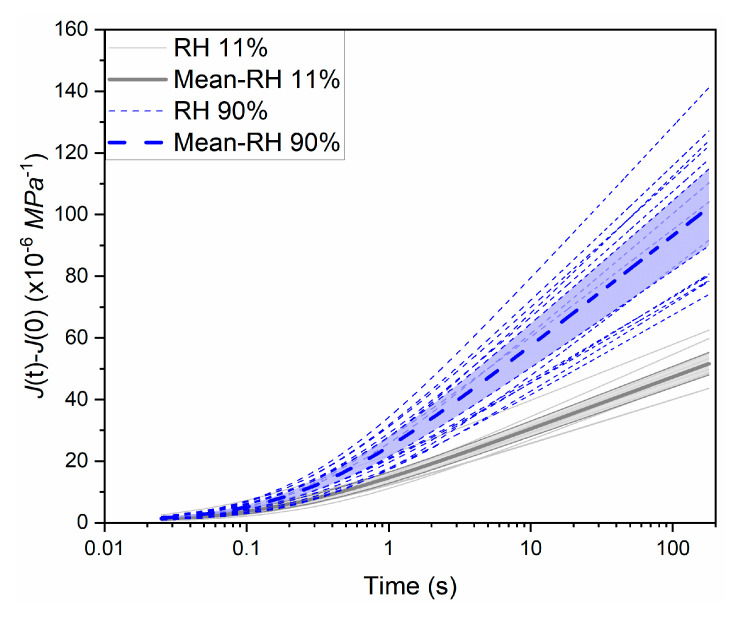
Time-dependent creep compliance at different RH levels: mean with 95% confidence interval (grey shading for RH 11% and blue shading for RH 90%).

In addition, ANOVA is carried out to examine the statistical significance of RH on the modeling parameters (C and $\tau_{0}$, α and β). These parameters appear to follow normal distributions as assessed by both the Anderson–Darling test and the Shapiro–Wilk test at the 0.05 significant level (Appendix B). As shown in Table 3, the *p*-values are 6.9 × 10^−10^, 0.005 for C and $\tau_{0}$, respectively, and 9.7 × 10^−6^, 0.002 for α and β, respectively, which are all lower than the significance level $\alpha_{0}$ = 0.05, all confirming the significant effects of RH. Further details of the ANOVA results are provided in Appendix A. Next, the creep compliance of microprism creep at different RH levels was also plotted and compared. As shown in Figure 9, all the creep compliances at the RH level of 90% are above those at the RH level of 11%, and the mean creep compliance at RH 90% is twice as large as that at RH 11%. Furthermore, we plotted the confidence interval (CI) of the compliance curves to examine the statistical significance of the RH effect. Here, $CI=\bar{x}\pm z\frac{s}{\sqrt{n}}$, $\bar{x}$ indicates the mean value, $\frac{s}{\sqrt{n}}$ denotes the standard error, and the z-value is 1.96 for a 95% confidence interval. It can be observed from the plot that there is no overlapping within the highlighted CI range at different RH levels. Generally, it can be concluded that the RH effect on the creep behavior of cement microprism is significant.

### 6.2. Comparison with Previous Findings of Microindentation Creep in Cement Paste

**Figure 10:**
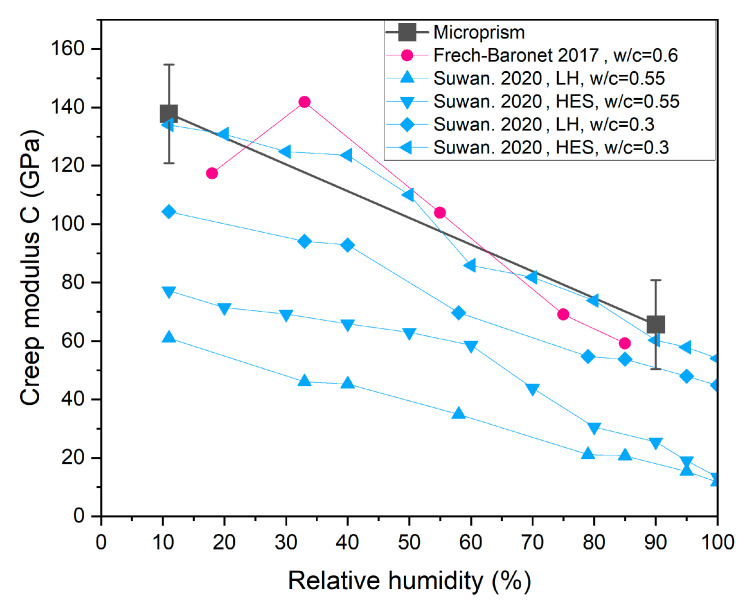
Comparison of creep modulus (C) measured by microprism compression and microindentation tests [25,26].

In this section, we compare the present results with previous findings obtained by microindentation with a similar probed volume but a different load configuration and stress level. While microprism creep can be viewed as uniaxial due to a ratio of $\sigma_{0}/f_{c}$ of 0.4, microindentation generates significantly higher confined stress levels, up to 400 MPa with an indentation depth of 30 μm [25,29], complicating modeling work to eliminate the plastic effect [28]. The material volume of the microprism (*V*_MP_) involved in the uniaxial compression creep, at 150 × 150 × 300 = 0.7 × 10^7^ μm^3^, is comparable in magnitude to the microindentation volume (*V*_MI_), which is approximately 1.4 × 10^7^ μm^3^, considering that indentation generates effects on the volume beneath the indenter 10 times those on the indentation depth. Therefore, it is interesting to compare the results of the two testing methods. As shown in Figure 10, our creep modulus C of the logarithmic model is compared with two previous studies on microindentation creep in cement paste: (i) where the w/c ratio was 0.6 and the RH level increased from 18% to 85% [25]; and (ii) where the w/c ratios were 0.3 and 0.55 for low-heat (LH) and high early strength (HES) cement, respectively, and the RH level decreased from 100% to 11% [26]. Generally, the creep modulus shows an approximately linear decreasing trend with increasing RH, regardless of the altering trend of RH levels. Thus, the creep modulus of microprisms in this study is consistent with those derived from microindentation tests in previous studies.

## 7. Conclusions

This work aimed to investigate the effect of internal water on the creep behavior of cement paste microprism under uniaxial compression. Based on the experimental and modeling results, the following conclusions can be drawn:

1. An analytical scheme was derived to address the foundation effect on the microprism creep. The derived coefficient $\varphi =(1+\frac{\sqrt{\pi }a(1-\nu^{2})}{2H}{)}^{-1}$, which decreases with an increasing ratio of a/H, aligns well with the FEM results. The foundation beneath the cement microprism accounts for approximately 30% of the total deformation observed in this study, which is non-negligible and should be excluded from the overall deformation.
2. Decreasing the RH level from 90% to 11% leads to notable increases in both the compressive strength ($f_{c}$) and Young’s modulus (E) of cement microprisms.
3. Both the logarithmic and power-law models were applied to analyze the creep. The presented results reveal a predominantly logarithmic nature of cement microstructure.
4. The RH has a significant effect on creep behaviors as determined by the ANOVA test. RH influences the fitting parameters C and $\tau_{0}$ in the logarithmic model, as well as α and β in the power-law model.
5. The time-dependent creep compliance at an RH level of 90% is approximately double that observed at an RH level of 11%.
6. The creep modulus C measured from microprism compression is consistent with previous results obtained using the microindentation technique.

While the current approach employs an elastic scheme to correct the foundation effect, future efforts can be focused on the viscoelastic theory to further evaluate this simplified method, given the viscoelastic nature of cement paste. Additionally, based on the findings from the two RH levels in this study, further explorations involving more RH levels may broaden the understanding of creep behaviors in cementitious materials.

## Figures and Tables

**Table 1:** Summary of the values of *f_c_* and *E* from the compression tests.

Microprism No.	RH 11%			RH 90%		
	$P_{max}$ (N)	$f_{c}$ (MPa)	E (GPa)	$P_{max}$ (N)	$f_{c}$ (MPa)	E (GPa)
MP-1	1.14	50.80	4.51	0.99	44.01	2.50
MP-2	1.82	80.68	4.19	1.08	48.12	4.10
MP-3	1.65	73.34	4.88	1.03	45.95	2.58
MP-4	1.75	77.71	2.90	0.84	37.53	2.66
MP-5	0.84	37.17	2.29	0.86	38.01	2.26
MP-6	1.40	62.33	4.30	1.33	59.20	3.00
MP-7	1.71	76.13	3.51	1.23	54.83	3.61
MP-8	1.34	59.67	4.16	1.20	53.27	3.46
MP-9	1.44	64.17	4.27	1.09	48.24	3.04
MP-10	-	-	-	0.97	43.31	3.56
MP-11	-	-	-	0.82	36.36	2.33
Mean ± Std Dev	1.45 ± 0.32	64.67 ± 14.20	3.89 ± 0.83	1.04 ± 0.17	46.26 ± 7.44	3.01 ± 0.60

**Table 2:** Modeling parameters in the power-law and logarithmic models.

RH	Power-Law			Logarithmic		
	α (GPa^−1^)	β	*R* ^2^	C (GPa)	$\tau_{0}$ (*s*)	*R* ^2^
11%	0.02 ± 0.003	0.19 ± 0.01	0.986 ± 0.004	137.8 ± 16.9	0.17 ± 0.07	0.999 ± 0.001
90%	0.04 ± 0.01	0.20 ± 0.01	0.991 ± 0.004	65.6 ± 15.3	0.26 ± 0.08	0.997 ± 0.002

**Table 3:** *p*-values of ANOVA for the fitting parameters at the significance level $\alpha_{0}$ = 0.05.

Power-Law		Logarithmic		$\alpha_{0}$
α	β	C	$\tau_{0}$	
9.7 × 10^−6^	0.002	6.9 × 10^−10^	0.005	0.05

## Data Availability

The original contributions presented in this study are included in the article. Further inquiries can be directed to the corresponding authors.

## Appendix A

The experimental data were analyzed through one-way ANOVA using the equations summarized in the table below.

**Table A1:** Key equations of ANOVA.

Source of Variation	Sum of Squares	Degree of Freedom	Mean Squares	*F* Statistic
Between groups	$SSB=\overset{k}{\underset{j=1}{\sum }}n_{j}(\bar{y_{j}}-\bar{y}{)}^{2}$	$df_{b}=k-1$	$MSB=SSB/df_{b}$	F=MSB/MSW
Within groups	$SSW=\overset{k}{\underset{j=1}{\sum }}\overset{n_{j}}{\underset{i=1}{\sum }}(y_{ij}-\bar{y_{j}}{)}^{2}$	$df_{w}=n-k$	$MSW=SSW/df_{w}$	-
Total	$SST=\overset{n}{\underset{i=1}{\sum }}(y_{i}-\bar{y}{)}^{2}$	$df_{t}=n-1$	-	-

Notes: n is the total number of observations; k is the number of groups; $n_{j}$ is the number of observations in group j; $\bar{y_{j}}$ is the mean of group j; $\bar{y}$ is the overall mean of all observations; $y_{ij}$ is the individual observation in group j.

The following tables present the ANOVA results at the two RH levels for compressive strength ($f_{c}$) and Young’s modulus (E), along with the logarithmic modeling parameters of creep modulus (C) and characteristic time ($\tau_{0}$), as well as the power-law modeling parameters of α and β.

**Table A2:** ANOVA results of compressive strength ($f_{c}$).

Source of Variation	*SS*	*df*	*MS*	*F*	*p-Value*
Between groups	1677.37	1	1677.37	13.94	0.0015
Within groups	2165.45	18	120.30	-	-
Total	3842.82	19	-	-	-

**Table A3:** ANOVA results of Young’s modulus (E).

Source of Variation	*SS*	*df*	*MS*	*F*	*p-Value*
Between groups	3.84	1	3.84	7.54	0.013
Within groups	9.17	18	0.51	-	-
Total	13.01	19	-	-	-

**Table A4:** ANOVA results of creep modulus (C).

Source of Variation	*SS*	*df*	*MS*	*F*	*p-Value*
Between groups	28,426.61	1	28,426.61	112.35	6.9 × 10^−10^
Within groups	5313.38	21	253.02	-	-
Total	33,739.99	22	-	-	-

**Table A5:** ANOVA results of characteristic time ($\tau_{0}$).

Source of Variation	*SS*	*df*	*MS*	*F*	*p-Value*
Between groups	0.053	1	0.053	9.97	0.005
Within groups	0.11	21	0.0053	-	-
Total	0.16	22	-	-	-

**Table A6:** ANOVA results of α for power-law modeling.

Source of Variation	*SS*	*df*	*MS*	*F*	*p-Value*
Between groups	0.0015	1	0.0015	33.45	9.7 × 10^−6^
Within groups	0.00095	21	4.53 × 10^−5^	-	-
Total	0.00245	22	-	-	-

**Table A7:** ANOVA results of β for power-law modeling.

Source of Variation	*SS*	*df*	*MS*	*F*	*p-Value*
Between groups	0.0017	1	0.0017	12.21	0.002
Within groups	0.0029	21	0.00014	-	-
Total	0.0046	22	-	-	-

## Appendix B

**Table A8:** Summary of normality test results at the significant level of 0.05: Shapiro–Wilk and Anderson–Darling tests.

Parameters	RH Level	Shapiro–Wilk Test		Anderson–Darling Test	
		Statistic	*p*-Value	Statistic	Critical Value
$f_{c}$	11%	0.927	0.449	0.303	0.693
	90%	0.953	0.682	0.223	0.680
E	11%	0.887	0.186	0.545	0.693
	90%	0.936	0.469	0.317	0.680
C	11%	0.902	0.232	0.495	0.684
	90%	0.888	0.091	0.609	0.679
$\tau_{0}$	11%	0.980	0.966	0.158	0.684
	90%	0.938	0.471	0.288	0.679
α	11%	0.871	0.101	0.479	0.684
	90%	0.886	0.087	0.661	0.679
β	11%	0.966	0.851	0.214	0.684
	90%	0.884	0.080	0.597	0.679
